# Functional Circuitry Effect of Ventral Tegmental Area Deep Brain Stimulation: Imaging and Neurochemical Evidence of Mesocortical and Mesolimbic Pathway Modulation

**DOI:** 10.3389/fnins.2017.00104

**Published:** 2017-03-03

**Authors:** Megan L. Settell, Paola Testini, Shinho Cho, Jannifer H. Lee, Charles D. Blaha, Hang J. Jo, Kendall H. Lee, Hoon-Ki Min

**Affiliations:** ^1^Department of Neurologic Surgery, Mayo ClinicRochester, MN, USA; ^2^Mayo Graduate School, Mayo ClinicRochester, MN, USA; ^3^Department of Surgery, Mayo ClinicRochester, MN, USA; ^4^Department of Physiology and Biomedical Engineering, Mayo ClinicRochester, MN, USA; ^5^Department of Radiology, Mayo ClinicRochester, MN, USA

**Keywords:** ventral tegmental area, deep brain stimulation, depression, functional magnetic resonance, fast-scan cyclic voltammetry

## Abstract

**Background:** The ventral tegmental area (VTA), containing mesolimbic and mesocortical dopaminergic neurons, is implicated in processes involving reward, addiction, reinforcement, and learning, which are associated with a variety of neuropsychiatric disorders. Electrical stimulation of the VTA or the medial forebrain bundle and its projection target the nucleus accumbens (NAc) is reported to improve depressive symptoms in patients affected by severe, treatment-resistant major depressive disorder (MDD) and depressive-like symptoms in animal models of depression. Here we sought to determine the neuromodulatory effects of VTA deep brain stimulation (DBS) in a normal large animal model (swine) by combining neurochemical measurements with functional magnetic resonance imaging (fMRI).

**Methods:** Animals (*n* = 8 swine) were implanted with a unilateral DBS electrode targeting the VTA. During stimulation (130 Hz frequency, 0.25 ms pulse width, and 3 V amplitude), fMRI was performed. Following fMRI, fast-scan cyclic voltammetry in combination with carbon fiber microelectrodes was performed to quantify VTA-DBS-evoked dopamine release in the ipsilateral NAc. In a subset of swine, the blood oxygen level-dependent (BOLD) percent change evoked by stimulation was performed at increasing voltages (1, 2, and 3 V).

**Results:** A significant increase in VTA-DBS-evoked BOLD signal was found in the following regions: the ipsilateral dorsolateral prefrontal cortex, anterior and posterior cingulate, insula, premotor cortex, primary somatosensory cortex, and striatum. A decrease in the BOLD signal was also observed in the contralateral parahippocampal cortex, dorsolateral and anterior prefrontal cortex, insula, inferior temporal gyrus, and primary somatosensory cortex (Bonferroni-corrected < 0.001). During neurochemical measurements, stimulation time-locked changes in dopamine release were recorded in the NAc, confirming that mesolimbic dopaminergic neurons were stimulated by DBS. In the parametric study, BOLD signal changes were positively correlated with stimulation amplitude.

**Conclusions:** In this study, the modulation of the neural circuitry associated with VTA-DBS was characterized in a large animal. Our findings suggest that VTA-DBS could affect the activity of neural systems and brain regions implicated in reward, mood regulation, and in the pathophysiology of MDD. In addition, we showed that a combination of fMRI and electrochemically-based neurochemical detection platform is an effective investigative tool for elucidating the circuitry involved in VTA-DBS.

## Introduction

Recently, deep brain stimulation (DBS) has been performed in several clinical trials to address treatment-resistant major depressive disorder (MDD) (Mayberg et al., 2005; Bewernick et al., 2012; Lozano et al., 2012; Schlaepfer et al., 2013; Dougherty et al., 2015). The evaluation of its efficacy and safety for the treatment of MDD is still in its first stages of development. However, there are several brain regions that have shown promising results as treatment targets of MDD with DBS (Anderson et al., 2012). The targeted brain regions include the subcallosal cingulate area (Mayberg et al., 2005; Lozano et al., 2012), the nucleus accumbens (NAc) (Bewernick et al., 2012), the ventral capsule/ventral striatum (that includes the NAc, olfactory tubercle, and islands of Calleja) (Dougherty et al., 2015), and the superolateral medial forebrain bundle (slMFB) (Schlaepfer et al., 2013). These structures, together with the ascending ventral tegmental area (VTA) mesocortical and mesolimbic pathways, play a crucial role in the regulation of mood, reward, and incentive-motivation processes that may be impaired in depressed individuals (Russo and Nestler, 2013).

The VTA, a central structure of the reward network, has been of interest in clinical research for MDD (Gazit et al., 2015; Furlanetti et al., 2016). This region contains the dopaminergic neurons of the mesocortical and mesolimbic circuitry, which project via the medial forebrain bundle (MFB) to the medial prefrontal cortex (mPFC), the NAc, the hippocampus, and the amygdala (Russo and Nestler, 2013). Such connections highlight the importance of the VTA in modulating mood and incentive-motivational behavior, and can thus, provide the theoretical basis for the stimulation of the VTA/MFB in rodent models of depression (Friedman et al., 2009; Bregman et al., 2015; Gazit et al., 2015; Furlanetti et al., 2016) and the slMFB for the clinical trials involving treatment-resistant MDD patients (Schlaepfer et al., 2013).

However, the VTA in its complex functional connectivity with many subcortical (striatum, thalamus, hippocampus, amygdala) and cortical structures (anterior cingulate, middle and inferior frontal gyri, parietal associative cortex, and insula) (Hadley et al., 2014), presents a need for a thorough evaluation of the stimulation effect on its functional circuitry. Dopamine-containing cells in the VTA project subcortically to the NAc, the amygdala, the hippocampus, the bed nucleus of the stria terminalis, the lateral septal area, the olfactory tubercle, and the lateral hypothalamus (collectively, these connections comprise the entire mesolimbic dopamine system), whereas separate dopamine-containing cells in the VTA project to cortical structures such as the prefrontal and insular cortex, and to a much lesser degree motor (M1) and related motor cortices (mesocortical dopamine system) (Berger et al., 1985; Oades and Halliday, 1987; Björklund and Dunnett, 2007; Friedman et al., 2009; Dichter et al., 2012; Russo and Nestler, 2013). Additional ascending dopaminergic projections from the VTA include the thalamus, hypothalamus, and the preoptic area (mesodiencephalic pathway) and the superior colliculus, reticular formation, periaqueductal gray, locus coeruleus, and cerebellum (mesorhombencephalic pathway) (Oades and Halliday, 1987). It is worth noting that many of these connections are reciprocal, in that that they also receive inputs from the same regions, as well as bilateral projections (Oades and Halliday, 1987).

Our group has recently used functional magnetic resonance imaging (fMRI) in a swine model to investigate the functional circuitry effect of DBS in the nucleus accumbens (Knight et al., 2013), another brain region targeted for the treatment of MDD. In the present study, we identified the functional connectivity by stimulating VTA and its surrounding mesolimbic and mesocortical structures: (1) We first combined DBS-fMRI and fast scan cyclic voltammetry (FSCV) in a within-subject large animal model (swine) study to confirm the activation of the mesocortical and mesolimbic dopaminergic pathways by measuring DBS-induced dopamine release in the NAc and investigated the functional circuitry effects of DBS in the VTA (VTA-DBS) in using fMRI [Repetition Time (TR): 3 s]; (2) Subsequently, in a second subject group, we performed higher-temporal resolution (TR 1.5 s) fMRI scans in a within-subject and within-scan study to confirm the sensitivity of the fMRI blood oxygen level-dependent (BOLD) response to several stimulation voltages.

Activating the VTA circuitry by DBS is a challenging task, and to date, there are few studies elucidating its functional connectivity in a large animal model. Our study provides an approach to identify the functional role of VTA-DBS and to observe how the VTA via the mesocortical and mesolimbic pathways modulate subcortical and cortical circuitry using fMRI techniques.

## Methods

### Subjects

**Figure 1:**
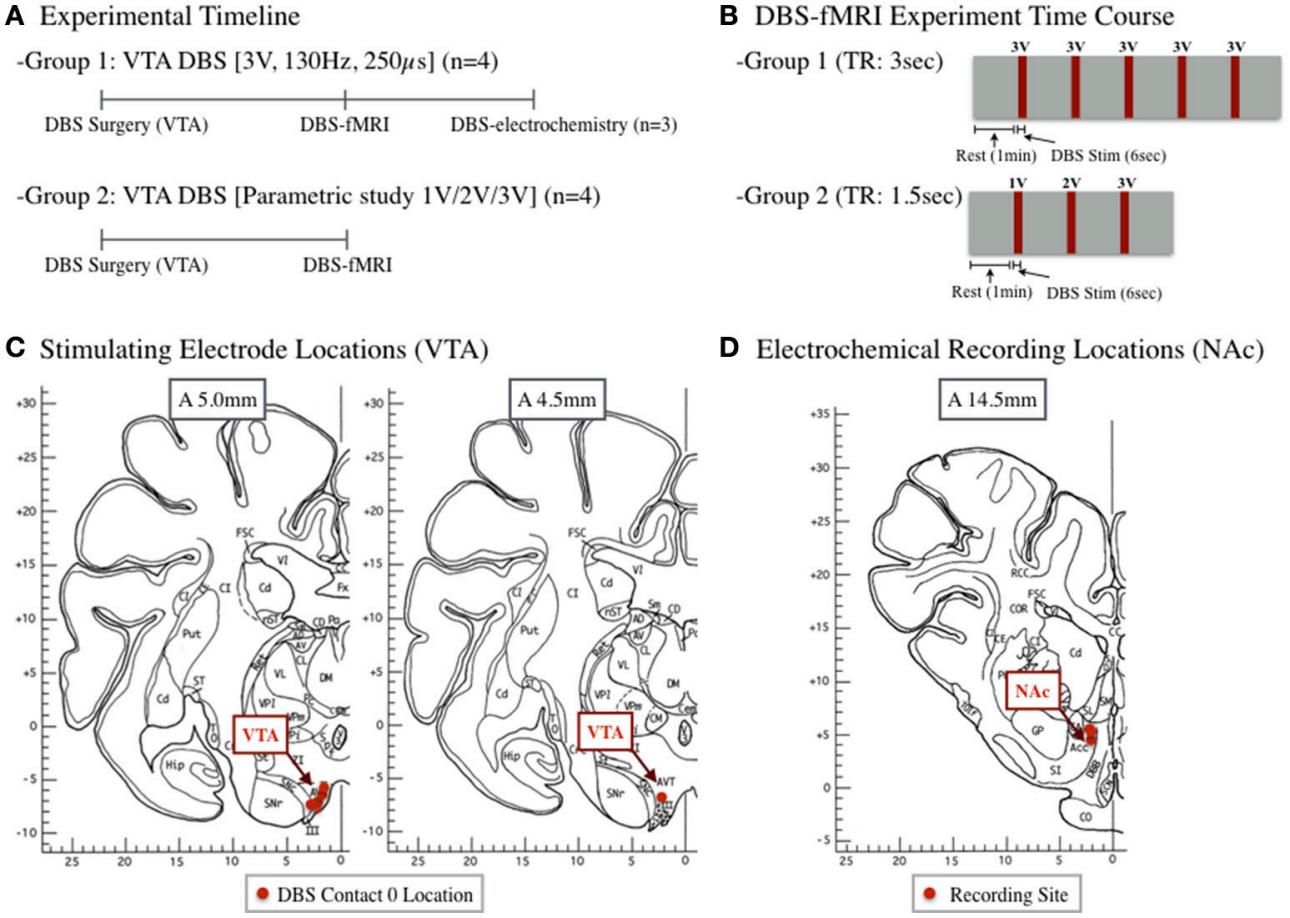
**(A)** A total of eight subjects underwent DBS surgery and had an electrode placed in the ventral tegmental area (VTA). Group 1 subjects (*n* = 4) underwent a standard stimulation protocol during fMRI, followed by subsequent electrochemistry (*n* = 3). A subset of subjects (Group 2, *n* = 4) underwent a parametric study following DBS surgery to evaluate the effects of varying amplitudes (1, 2, 3 V). **(B)** Group 2 subjects underwent a stimulation block design of 1 min rest, followed by a 6 s, 3 V stimulation, repeated 5 times. Group 2 design included a 1 min rest, followed by a 6 s, 1, 2, or 3 V stimulation. **(C)** Location of VTA stimulating electrode (contact 0). **(D)** Location of electrochemical recording electrode in the nucleus accumbens of each subject. Pig brain atlas used from Félix et al. (1999).

All study procedures were performed in accordance with the National Institutes of Health Guidelines for Animal Research (Guide for the Care and Use of Laboratory Animals) and approved by Mayo Clinic Institutional Animal Care and Use Committee. The subject group consisted of 8 normal healthy domestic swine (30 ± 3 kg). Animals were housed individually in a controlled environment (humidity 45% and temperature 21°C) and were fed once a day, with *ad libitum* access to water. The detailed study design and groups are described in Figure 1A.

### DBS surgery

DBS electrode targeting and implantation was performed with an MR image-guided Leksell stereotactic targeting system (Elekta Inc., Stockholm, Sweden) modified for large animals (Min et al., 2012; Kim et al., 2013). A 3 Tesla MR scanner (General Electric Healthcare, Wakasha, WI; Signa HDx, 16x software) with a custom four-channel transmit-receive radiofrequency coil was used for acquiring MR images. 3D magnetization prepared rapid acquisition gradient echo (MPRAGE) images were used for MR image-based targeting with swine brain atlas (Félix et al., 1999; Saikali et al., 2010) and COMPASS navigational software (Stereotactic Medical Systems, Rochester, MN), modified for large animals, to determine the Leksell coordinates for stimulation target (Min et al., 2012).

Sedation was maintained with 1.5–3% isoflurane during surgery and 1.5–1.75% isoflurane during the fMRI and NAc dopamine recording experiments. Vital signs were continuously monitored throughout all the procedures. Upon sedation, subjects were implanted with a quadripolar (contacts labeled 0, 1, 2, and 3) DBS electrode (Model 3389, Medtronic, Inc.). The electrode (contact 0) position targeted to the VTA are shown in Figure 1C, based on the initial subject specific MR image and targeting coordinates (Min et al., 2012).

### Fast-scan cyclic voltammetry

As described by Min et al. (2016), FSCV recordings were obtained using a 7 μm diameter, ~100 μm length carbon fiber sensing electrode. The sensing electrode was targeted toward NAc based on subject specific MR brain images. Dopamine signals were recorded by either applying a triangular waveform (−0.4, 1.5, −0.4 V) or an N-shaped waveform (−0.4, 1.0, −0.4, −1.4 V). Dopaminergic signals were recorded across the three subjects in Group 1. (As a preliminary setup, one animal in Group 1 was used to initially conduct only the DBS-fMRI part and not followed by a FSVC study). Changes in dopamine oxidation current signal at +0.6 V in the NAc in response to VTA-DBS (130 Hz frequency, 0.25 ms pulse width, 3 V amplitude, and 2 s stimulation) was normalized to the average background current for each subject (Min et al., 2016). The dopamine sensing area in NAc was marked in Figure 1D based on the initial subject specific MR image and targeting coordinates (Min et al., 2012).

### Functional MRI

Stimulation during fMRI acquisition (gradient echo, echo-planar imaging pulse sequence) consisted of two distinct protocols as shown in Figure 1B (group one: 130 Hz frequency, 0.25 ms pulse width, and 3 V amplitude; group two: 130 Hz, 0.25 ms, and increasing amplitudes of 1, 2, and 3 V). The scanning consisted of a block design to detect changes in BOLD shown in Figure 1B. Data processing and analyses were completed as previously described (Min et al., 2012). Briefly, fMRI data was converted to BrainVoyager format (Brain Innovation, BrainVoyager QX, Netherlands) and a standard sequence of pre-processing steps was applied to each subject (slice time correction, motion correction, special smoothing, and temporal filtering). Functional activation maps (t-maps) were generated using a double-gamma hemodynamic response function, each representing the block design for the corresponding voxel.

Each voxel was then, registered into swine brain atlas space and group analyses were computed using a fixed-effects analysis to concatenate the data from all the patients. We integrated the multiple-subject data into a single general linear model analysis. To correct for false positive voxels, we implemented multiple comparisons and only included voxels with a Bonferroni corrected *p* < 0.001 as regions of interest with significant changes in BOLD (Min et al., 2012).

To obtain a quantitative estimate of the correlation between stimulation voltage and the magnitude of regional hemodynamic response of subjects in group 2, we conducted a linear regression between the voltage intensity and the BOLD % change. Linear regression was applied to the peak values of BOLD percent change across three voltage levels (1, 2, and 3 V). The slopes, intercepts, and R2 values of the linear model were obtained from the eight regions of interests (ROIs) in each individual subjects. These values were averaged and tested for statistical significance (one sample *t*-test). We calculated the two-factor ANOVA (subject × voltage) and performed multiple comparisons to identify how each factor could help explain the differences we observed in BOLD responses upon different stimulation voltages and subject-dependent variability.

## Results

### Confirmation of NAc and VTA connectivity

**Figure 2:**
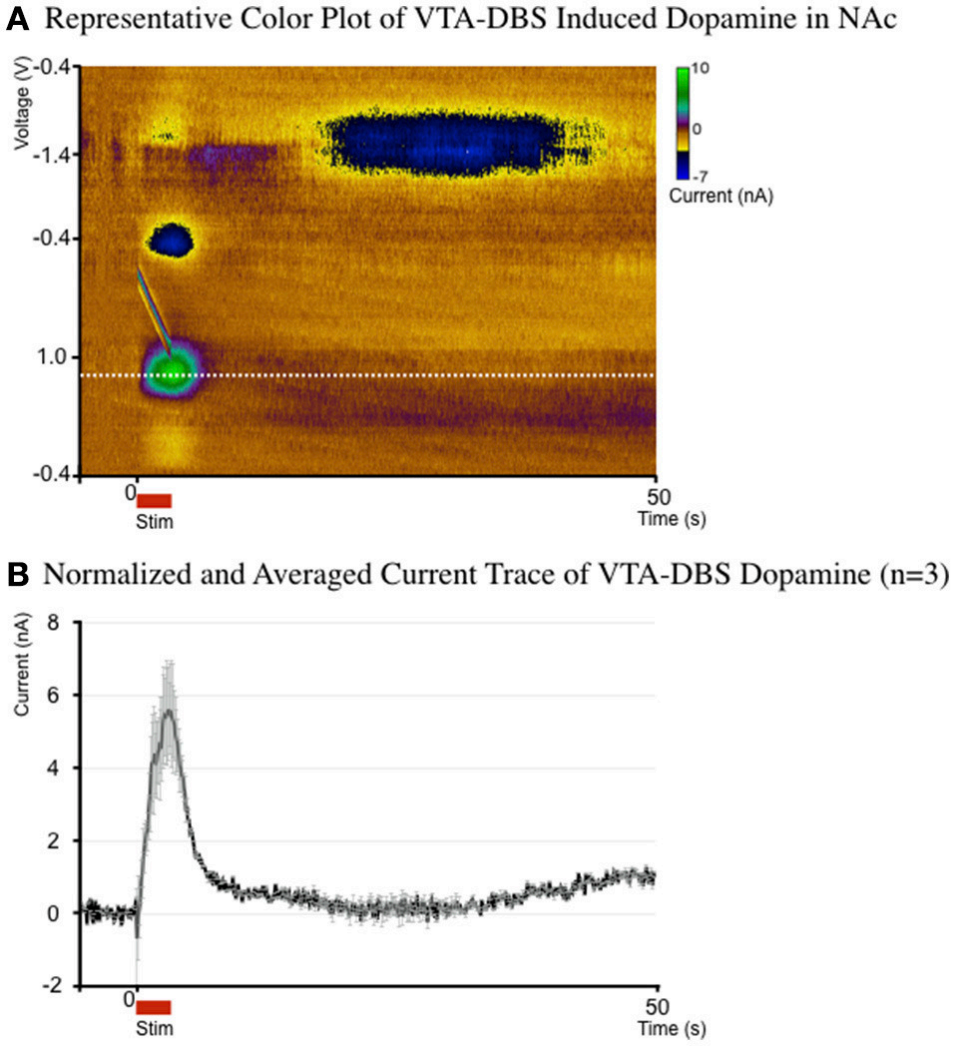
**(A)** Representative pseudo-color plot of dopamine oxidation (0.6 V) and reduction (−0.2 V) following VTA-DBS. **(B)** The average (± standard error) current by time trace of a single representative dopamine response, at the dopamine oxidation voltage for the 3 subjects who underwent electrochemical evaluation (normalized to the average background current).

The VTA contains dopaminergic projections that target the NAc via the MFB, leading to a dopaminergic electrochemical signature in the NAc during VTA-DBS. Figure 2A is a representative pseudo-color plot, showing the electrochemical signature of dopamine, oxidation (+0.6 V) and reduction (−0.2 V) following VTA-DBS. In addition, Figure 2B shows the time course and magnitude of changes in normalized and averaged dopamine oxidation currents at +0.6 V evoked by VTA-DBS (*n* = 3). Individual dopamine results included in Supplementary Figure 1.

### fMRI BOLD signaling during DBS in the VTA

**Figure 3:**
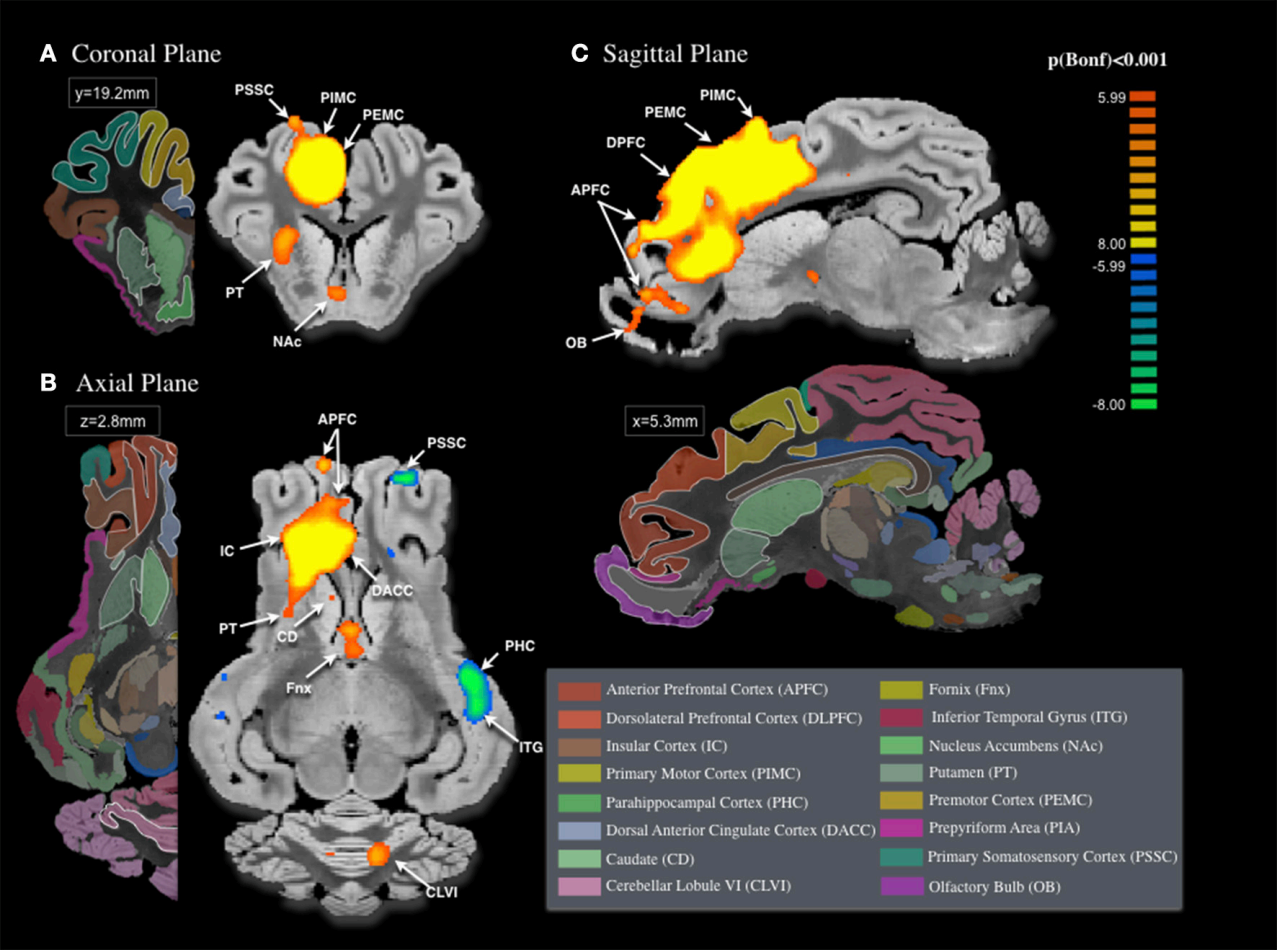
**Areas of activation with unilateral ventral tegmental area (VTA) stimulation (3 V, 250 μs, 130 Hz, *n* = 4)**. Areas of significant BOLD change in the **(A)** Coronal Plane, **(B)** Axial Plane, and **(C)** Sagittal Plan included cortical structures [anterior prefrontal cortex APFC, dorsolateral prefrontal cortex DLPFC, primary motor cortex PIMC, dorsal anterior cingulate cortex DACC, premotor cortex PEMC, and primary somatosensory cortex PSSC, insular cortex IC, and parahippocampal cortex PHC (contralateral)], subcortical structures [caudate CD, fornix Fnx, inferior temporal gyrus ITG (contralateral), nucleus accumbens NAc, putamen PT, prepyriform area PIA, olfactory bulb OB and cerebellar lobule VI CLVI (contralateral). All areas of activation deemed significant were Bonferroni-corrected, *p* < 0.001]. 3D pig brain atlas template used from Saikali et al. (2010).

The fMRI BOLD signal changes induced by the stimulation of VTA, demonstrated in Figure 3, were significant across multiple brain structures of the mesolimbic and mesocortical pathways. During VTA-DBS, we observed significant BOLD signal changes in both the ipsilateral and contralateral hemispheres (Bonferroni correction < 0.001). This included the striatum, associative cortex (anterior prefrontal cortex), limbic structures (insula, dorsolateral prefrontal cortex, prepyriform area, dorsal anterior and posterior cingulate cortex, NAc, hippocampus, inferior temporal gyrus, parahippocampal cortex, and perirhinal cortex), and the sensorimotor networks (premotor, primary motor and primary somatosensory cortices, and cerebellum).

We found that the most significant BOLD changes were located in the premotor (*z* = 16.96), primary motor cortices (*z* = 15.62), dorsal posterior cingulate cortex (*z* = 15.60), and dorsolateral prefrontal cortex (*z* = 14.93) on the ipsilateral side of stimulation, as shown in Table 1. Bilateral changes in BOLD signal were located in the associative, insular, limbic, and sensory-motor regions. In the anterior prefrontal cortex, insula and anterior cingulate cortex, we observed an ipsilateral increase (*z* = 14.93, *z* = 14.26, and *z* = 13.86, respectively), and a contralateral decrease (*z* = −8.91 and *z* = −9.11, respectively) in BOLD signal. Significant changes in BOLD signal were observed in the limbic structures, including the dorsolateral prefrontal cortex (ipsilateral: *z* = 14.93; contralateral: *z* = −7.47) and prepyriform area (ipsilateral: *z* = −9.26; contralateral: *z* = −10.84), and in the primary somatosensory cortex (ipsilateral: *z* = 14.24; contralateral: *z* = −8.72). Additionally, ipsilateral increases in BOLD signal were mainly located in the limbic regions (dorsal anterior cortex: *z* = 13.86; dorsal posterior cortex: *z* = 15.60), NAc (*z* = 7.97), caudate (*z* = 6.31), and putamen (*z* = 11.47). In contrast, the perirhinal cortex showed negative BOLD change (*z* = −6.73). To check individual variability, probabilistic map included in Supplementary Figure 2.

**Table 1:** **Brain areas of significant BOLD response across Group 1 subjects**.

**Networks**	**Ipsilateral**	**Size (mm^3^)**	***Z*-score**	**Contralateral**	**Size (mm^3^)**	***Z*-score**
Associative	Anterior prefrontal cortex (I)	1,050	13.83	Anterior prefrontal cortex (I)	31	−8.91
	Dorsolateral prefrontal cortex (I)	771	14.93	Dorsolateral prefrontal cortex (I)	44	−9.26
Limbic	Prepyriform area (I)	54	−7.47	Prepyriform area (I)	64	−10.85
	Insular cortex	119	14.26	Insular cortex	49	−9.11
	Dorsal anterior cingulate cortex (I)	442	13.86			
	Dorsal posterior cingulate cortex (I)	167	15.60			
	Nucleus Accumbens (I)	94	7.97			
	Perirhinal cortex (I)	3	−6.73			
				Fornix and hippocampus (C)	33	7.69
				Inferior temporal gyrus (C)	72	−9.11
				Parahippocampal cortex (C)	44	−9.72
Sensory/motor	Primary somatosensory cortex (I)	900	14.24	Primary somatosensory cortex (I)	142	−8.72
	Premotor cortex (I)	1,041	16.96			
	Primary motor cortex (I)	230	15.62			
				Cerebellar lobule V (C)	42	7.58
				Cerebellar lobule VI (C)	59	7.62
				Cerebellar lobule VIIB (C)	29	7.6
Basal ganglia	Caudate (I)	2	6.31			
	Putamen (I)	169	11.47			

*Associative network activation included the anterior prefrontal cortex on both the ipsilateral and contralateral side. Limbic regions of significant BOLD change included dorsolateral prefrontal cortex, prepyriform area, insular cortex (bilateral), dorsal anterior cingulate cortex, dorsal posterior cingulate cortex, nucleus accumbens, perirhinal cortex (ipsilateral), fornix and hippocampus, inferior temporal gyrus, parahippocampal cortex (contralateral). Sensory/motor regions included primary somatosensory cortex (bilateral), premotor and primary motor cortex (ipsilateral), and cerebellar lobules V, VI, VIIB (contralateral). Basal ganglia regions included ipsilateral caudate and putamen*.

### The effect of varying simulation voltage on BOLD signaling during DBS

Our results also revealed a positive relationship between stimulation voltage and fMRI BOLD signal. We applied three different stimulation voltage levels (1, 2, and 3 V) to test the effect on the differential activation patterns in several regions of the brain.

**Figure 4:**
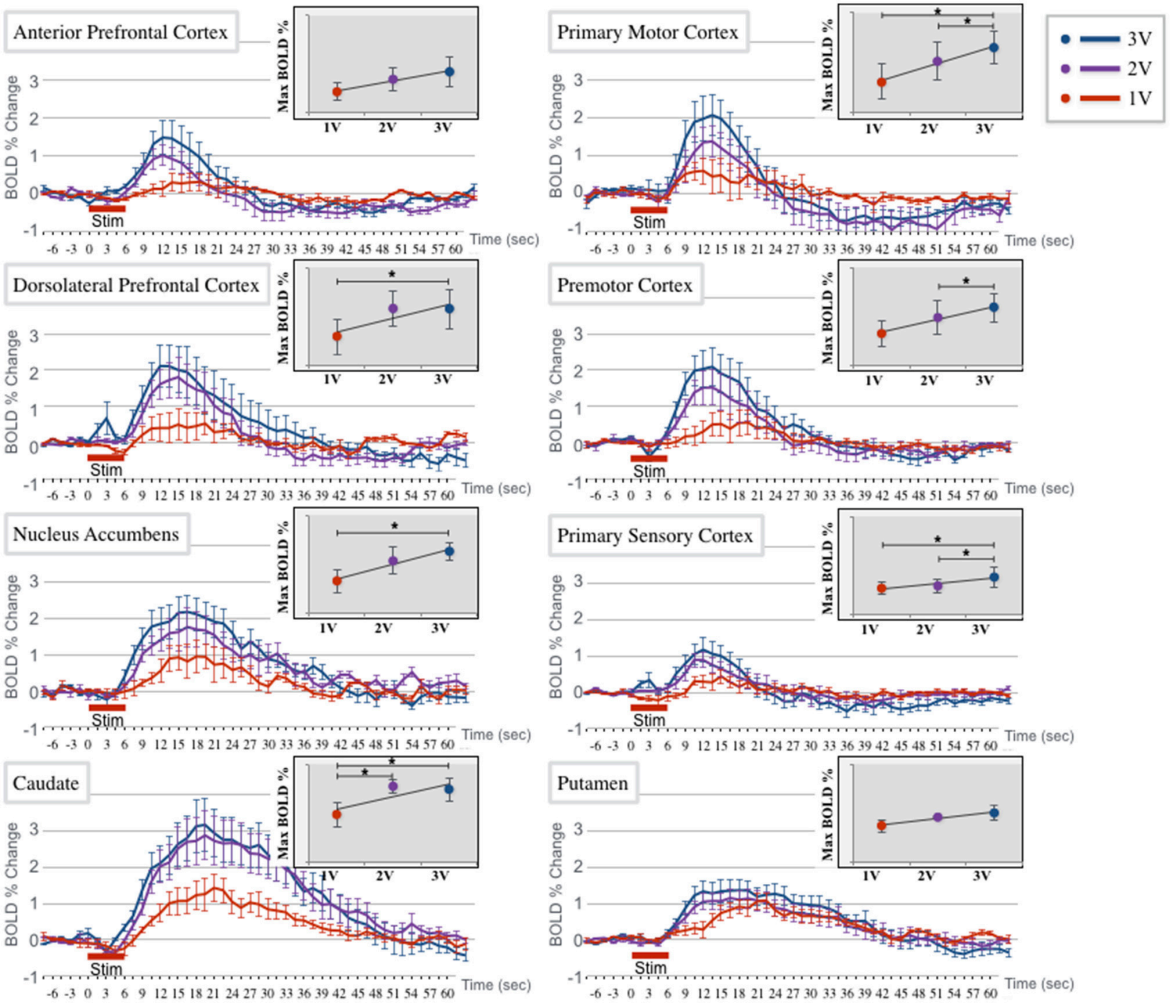
**BOLD % change time course at 1, 2, and 3 V stimulation amplitude (red, purple, and blue, respectively) at each of 8 regions of interest (ROI): anterior prefrontal cortex (APFC), primary motor cortex (PIMC), dorsolateral prefrontal cortex (DLPFC), premotor cortex (PEMC), nucleus accumbens (NAc), primary somatosensory cortex (PSSC), caudate (Cd), and putamen (PT)**. Each amplitude is presented as the average (± standard error) across the 3 subjects in each region of interest (ROI). Insert: The inset graph demonstrates a linear regression of the peak value at each of the three amplitudes, with significant differences denoted with a star (^*^). The APFC, DLPFC, PEMC, NAc, and Cd all had significant differences in BOLD percent change between 1 and 3 V amplitudes. APFC also showed a significant difference between the 2 and 3 V amplitudes. PIMC, PSSC, and PT showed no significance between any of the three amplitudes.

We found a positive trend in the BOLD signal changes as the stimulation voltage increased (Table 2). The linear model with group averaged slopes and intercepts were plotted in Figure 4 for each of eight ROIs separately. The slopes of linear model measured in the caudate (*T* = 9.8, *p* = 0.002), dorsolateral prefrontal cortex (*T* = 3.9, *p* = 0.03), nucleus accumbens (*T* = 3.6, *p* = 0.04), primary motor cortex (*T* = 4.6, *p* = 0.02), and primary somatosensory cortex (*T* = 4.1, *p* = 0.03) were significant with the statistical threshold at *p* < 0.05. There was no statistical significant of slope in other regions, but showed positive trends.

**Table 2:** **Summary table of linear regression model between voltage and BOLD change**.

**Regions**	**Slope**	**Intercept**	***p*-value**
APFC	0.33	−0.16	0.11
CD	0.50	0.80	0.01^**^
DLPFC	0.40	0.07	0.03^*^
NAc	0.39	0.50	0.04^*^
PIMC	0.38	−0.19	0.02^*^
PEMC	0.30	0.00	0.11
PSSC	0.23	0.12	0.03^*^
PT	0.51	0.55	0.28

The slopes and intercepts of four subjects were averaged for each of the eight region-of-interests (ROIs). The test of significance was conducted by one-sample t-test on the slope of the linear model. (

* *P < 0.05*,

** *P < 0.01)*.

Two-factor ANOVA (subject × voltage) indicated a significant difference (*p* < 0.05) in BOLD response when induced by three different voltage levels in eight ROIs. We found the significant effect of voltage levels in the ROIs (*F* > 5.2, *p* < 0.05), except the nucleus accumbens, premotor cortex, and putamen. In multiple comparisons of *post-hoc* analysis, the sensorimotor cortices (primary motor cortex, and primary somatosensory cortex) showed significant differences (*p* < 0.05) in BOLD response between 1 and 3 V, while the caudate showed differences between the 1 and 2 V, and between 1 and 3 V stimulation. We also observed considerable inter-subject variability in BOLD response across the three voltage levels. In all the ROIs except the putamen, significant BOLD response differences between subjects were observed (*F* > 4.7, *p* < 0.05). The regression results indicated that the significant inter-subject variability was in part due to the difference in the intercept (for details please see Supplementary Table 1).

## Discussion

### Functional connectivity of the VTA

Due to the complexity of the VTA circuitry and its extensive connections to a variety of cortical and subcortical brain regions, the full neuromodulatory effect of stimulating VTA or other nodes within its circuitry is still poorly understood. To better understand the global connectivity and the pattern of NAc dopamine release induced by electrical stimulation of the VTA and surrounding structures, we combined FSCV and fMRI techniques and applied them to a large animal (swine) model of VTA-DBS to study the high temporal resolution fMRI BOLD responses and its links the stimulation voltage sensitivity.

Our fMRI results suggest that VTA-DBS directly modulates the activity of a subset of regions anatomically and functionally connected to the VTA. Upon VTA stimulation, we observed BOLD signal changes in the bilateral dorsolateral prefrontal cortex and the ipsilateral posterior cingulate. These cortical regions, adjacent to the parahippocampal gyrus, inferior temporal cortex, and insula, are involved in working memory, arousal and awareness, and the regulation of mood, emotion, and reward (Pochon et al., 2002; Leech and Sharp, 2014). It is in these regions that depressive patients show alterations in neural activity (Drevets et al., 2008).

The present data also show BOLD changes in several of the mesostriatal components, such as the caudate, putamen, and fornix. Interestingly, as shown in Table 1, the majority of negative BOLD change was localized to the contralateral side of the stimulation, including the dorsolateral prefrontal and insular cortex, as well as the anterior prefrontal and primary somatosensory cortex. Additionally, our data demonstrate that a few areas, not traditionally associated with the VTA circuit, were activated during stimulation. These areas include the parahippocampal cortex, inferior temporal gyrus, and prepyriform area.

Our current understanding for how DBS affects the structures surrounding the VTA, such as the substantia nigra (SN), located immediately lateral to the VTA, is still very limited. Like the VTA, the SN also projects to the motor and associative striatum (caudate and putamen) (Joel and Weiner, 1997), and to the subthalamic nucleus (Parent and Hazrati, 1995). Both structures also contain projections to the cingulate and frontal cortex (Fallon and Moore, 1978; Pioli et al., 2008). Therefore, this overlap between the SN and VTA projections presents a potential implication to our study. Although, our goal was to specifically target the VTA, it is possible that, depending on the electrode placement, the effect of the stimulation could have spread to the SN and dopamine-containing cells therein comprising the dopaminergic nigrostriatal pathway. Since both VTA and SN fibers run through the MFB, it is necessary to confirm whether the SN stimulation was the result of an epiphenomenon of VTA stimulation before we assess the clinical efficacy and its short- or long-term effects (e.g., hyperdopaminergic stimulation).

### VTA network implicated in MDD

The central role of the VTA in the limbic and reward network is of relevance in MDD condition, because VTA dopamine-containing cells project to key areas including the cingulate cortex, medial prefrontal cortex, NAc, hippocampus, amygdala, olfactory tubercle, and entorhinal and pyriform cortices (Björklund and Dunnett, 2007; Friedman et al., 2009; Russo and Nestler, 2013). Human fMRI studies have found that during a reward task, the VTA and NAc BOLD responses were positively correlated, suggesting a functional connection between the two areas (D'Ardenne et al., 2008). Additional fMRI studies further confirmed that the reward-processing regions, like the striatum (caudate and putamen), medial prefrontal cortex, pregenual and subgenual anterior cingulate, and medial frontal gyrus, were hypoactive in MDD patients (Dichter et al., 2012). The VTA projections to the NAc continue to be of importance in MDD research because of their role in modulating the effect of excitatory glutamatergic inputs that originate from the limbic (e.g., amygdala and ventral subiculum of the hippocampus) and prefrontal cortex (Dichter et al., 2012).

The areas strictly implicated in MDD include the prefrontal cortex (medial, dorsolateral, orbital, and ventromedial frontal polar), cingulate (dorsal and subgenual anterior, and dorsal posterior), temporopolar cortex, premotor cortex, ventral striatum (including the NAc), amygdala, parahippocampus, and medial thalamus (Drevets and Raichle, 1992; Mayberg et al., 2000, 2005; Neumeister et al., 2004; Drevets et al., 2008; Hasler et al., 2008; Anderson et al., 2012; Singh and Gotlib, 2014). Different studies report contrasting views on the patterns of hypo- or hyperactivity in these regions in MDD patients. Although, the reason for this phenomenon is unclear, it has been suggested that different compensatory mechanisms may be present in the depressive state in different patients (Mayberg, 2003). Additionally, the pattern of activation in the dorsal and subgenual anterior cingulate and amygdala was found to be a predictor of response to psychotherapy and pharmacological treatment (Singh and Gotlib, 2014).

MDD patients who were responsive to pharmacological treatments revealed decreased activity in the cingulate (subgenual, anterior, and posterior), prefrontal (medial and orbital), parietal (precuneus, inferior parietal lobule), and temporal cortices, amygdala, hippocampus, parahippocampus, pallidum, insula, and habenula; and increases in the prefrontal (dorsolateral, dorsomedial, ventrolateral) cortex, anterior and posterior cingulate, insula, and parietal cortex (Mayberg et al., 2000; Kennedy et al., 2001; Singh and Gotlib, 2014). Those who were responsive to interpersonal psychotherapy and cognitive behavioral therapy were associated with modulatory activity in the prefrontal cortex, hippocampus, and anterior cingulate (Brody et al., 2001; Goldapple et al., 2004). So far, the effects of treatment over subgenual and posterior cingulate activity have been the most relevant in connection with clinical improvement (Mayberg, 2003).

As a follow-up study, researchers applied stimulation to the subgenual cingulate, a known target to the VTA, in patients with treatment-resistant MDD (Mayberg et al., 2005). Initially, the researchers tested the efficacy of this treatment with objective markers and next performed positron-emission tomography (PET) in the treated patients pre- and postoperatively (Mayberg et al., 2005). In the responders, the changes in activation pattern revealed similarities with those associated with pharmacological and behavioral therapies. Metabolism was reduced in the orbital and medial frontal cortices, hypothalamus, and insula, and increased in the dorsolateral prefrontal, premotor, and parietal cortices, and in the dorsal anterior and posterior cingulate (Mayberg et al., 2005; Lozano et al., 2008). PET data collected in patients undergoing NAc-DBS for MDD observed activity changes compared to the preoperative activity levels in many of these areas (Bewernick et al., 2010). The induction of the NAc with DBS resulted in a decreased activity in the orbital prefrontal cortex, subgenual and posterior cingulate, thalamus, and caudate; increased activity in the precentral gyrus; and decreased amygdala metabolism only in responders (Bewernick et al., 2010).

Sensorimotor cortex activity was also affected by VTA-DBS. The VTA output to the bilateral primary motor cortex is important for motor skill training and, therefore, is currently an area of interest in motor rehabilitative medicine (Kunori et al., 2014). As noted above, neuromodulation of the sensorimotor network was likely mediated by current spread to the SN, which entails a higher level of connectivity with the sensorimotor network (Kwon and Jang, 2014). While premotor cortex activity changes have been associated with both MDD and with its response to treatment, we cannot conclude whether the BOLD signal change we observed in the primary motor cortex specifically plays a role in VTA-DBS for MDD or is a result of a epiphenomenon of the connectivity between these regions.

### Dopaminergic and non-dopaminergic network involved with VTA/MFB DBS

While we aimed to confirm dopamine release induced in the NAc by VTA-DBS, other neurotransmitters are known to be involved in the complex VTA circuitry. Both dopamine and serotonin precursors (Nakahara et al., 2000) as well as dopamine itself (Hernandez et al., 2006) are present in the NAc, and their concentrations change during intracranial self-stimulation experiments, in which the MFB is stimulated to evoke hedonic effects. Additionally, VTA-DBS evoked BOLD response appears to be mainly glutamatergic dependent (Helbing et al., 2016), suggesting that the effects of VTA-DBS are mediated by a combination of dopaminergic and non-dopaminergic networks. Interestingly, in this study (Helbing et al., 2016) dopamine played a synergistic role with glutamate in eliciting BOLD signal changes and was especially relevant in the setting of continuous stimulation. This is discordant with evidence that intermittent but not continuous stimulation works by dopaminergic activity modulation (Bregman et al., 2015). MFB self-stimulation in rodents is known to induce dopamine increase in the NAc (Nakahara et al., 1992). Self-stimulation consists in intermittent stimulation and is a paradigm more representative of reward and addiction than of anti-depressive activity, vs. continuous stimulation, which mimics the therapeutic model represented by DBS (Bregman et al., 2015). In a recent study in rodents, the antidepressant effect of continuous MFB-DBS *in vivo* at stimulation parameters clinically relevant was mediated neither by dopamine nor by serotonin release in the NAc (Bregman et al., 2015). It is therefore possible that the dopamine signal observed in our study would not be recorded with continuous stimulation.

### Adverse effects of differential neuromodulation of VTA

Lastly, we evaluated the differential neuromodulation effects induced by the increase of stimulation voltages during the delivery of VTA-DBS. A recent study investigating VTA-DBS in patients with cluster headaches reported adverse effects at high voltages (Akram et al., 2016). At higher voltages, patients experienced tachycardia, raised blood pressure, vertical diplopia and feelings of panic. VTA-DBS appeared to differentially modulate the global neural activity in presence of high stimulation voltage, resulting in the adverse effects. However, despite these adverse effects, changes in stimulation voltage continue to be the prominent approach to deliver the therapeutic effect of DBS in PD patients (Moro et al., 2002).

These symptoms bring to surface the importance of establishing an objective marker to assess the differential neuromodulatory effects of stimulation at the varying voltages. There seems to be a ceiling effect to the therapeutic benefits of DBS, where beyond a certain point, the detrimental side effects appear (Gibson et al., 2016a). Adverse effects observed in clinical trials, including hypomania, mania, and disinhibition (Malone et al., 2009; Bewernick et al., 2010; Dougherty et al., 2015) are comparable to the effects elicited by drugs of abuse in VTA-DBS animal studies (Cleary et al., 2015).

### FSCV and fMRI application for future VTA-DBS

Our dual approach, using FSCV and fMRI, may be an effective way to observe objectively brain network changes evoked by DBS. Increases in the stimulation voltages have been associated with increased BOLD percent change and larger cluster sizes, suggesting that a larger electrical spread is able to recruit more cell bodies and/or axonal fibers (McIntyre and Hahn, 2010; Knight et al., 2013; Paek et al., 2015). These findings, along with our results, demonstrate data showing that fMRI could be used as an indicator for the global effect of VTA-DBS on mesolimbic and mesocortical circuitry. It presents a potential way of addressing the risks associated with stimulation of the reward circuitry for those individuals considered for future DBS procedures. It is evident that the connectivity of the VTA circuit is complex and may provide us with an approach to address current MDD symptomatology through widespread neuromodulation. However, with such structures with wide influences within the brain, it would be crucial to approach with scientific awareness of the risks associated with manipulating these structures.

### Limitations

The small sample size (*n* = 4 per group) limits our statistical power to conclude any generalizations from our results. To control for DBS targeting error and variability, we performed precision MR imaged guided stereotactic surgery, identical to the human DBS surgery. We have previously shown, despite the small sample size, consistent results with high statistical power (Paek et al., 2015; Gibson et al., 2016b; Ross et al., 2016). In our current study, we also applied Bonferroni correction to exclude false positive results.

We recognize that the anesthetized and non-disease state of the animal prose limitations in our interpretation of our data. We have previously compared results in a small number of Parkinson's patients in the awake state (*n* = 5) and the anesthetized state (*n* = 5; Knight et al., 2015). We have also reported a study involving patients with essential tremor, confirming the functional correlates of the therapeutic and adverse effects evoked by thalamic stimulation using fMRI (Gibson et al., 2016a). The DBS-fMRI data was collected during the anesthetized state, and then compared with data from the awake-clinical state in the tremor patients. We believe our study demonstrates an effective technique that could be useful for functional network mapping studies for DBS in the near future, possibly in combination with other behavioral tests and animal disease models.

## Conclusions

In this study, we aimed to characterize the modulation of the neural circuitry associated with VTA-DBS in a large animal. Our findings suggest that VTA-DBS affects the activity in areas implicated in working memory, arousal and awareness, reward, mood regulation, and the pathophysiology of MDD. VTA-DBS therefore affects the function of circuits potentially related to the symptoms of treatment-resistant MDD. Further studies in animal depression models and patients with MDD will be necessary to confirm these results and improve the array of therapeutic options in treatment-resistant mood disorders.

## Author contributions

Designed model framework: MS, PT, HJ, KL, HM. Performed experiments: MS, HM. Analyzed the data: MS, SC, HJ, HM. Wrote the manuscript: MS, PT, SC, JL, CB, HM.

### Conflict of interest statement

The authors declare that the research was conducted in the absence of any commercial or financial relationships that could be construed as a potential conflict of interest.

## Supplementary material

Supplementary Figure 1 **Individual dopamine results**.

Supplementary Figure 2 **To check individual variability, probabilistic map included made with individual FDR < 0.05 activation maps**.

Supplementary Table 1 **Summary of coefficients for individual linear regression analysis**. The table shows the regression coefficients of the voltage changes (1, 2, and 3 V) on the percent change of BOLD peaks by region-of-interests (ROIs) for each of the four subjects. Three parameters (slope, intercept, and coefficient determination—*R*^2^) were estimated separately for each subject. The *R*-square value of the individual indicates the proportion of the within-subject variance in BOLD % change, predicted from the voltage changes. *R*^2^ demonstrates the tendency of inter-subject variability in voltage-dependent effects. Linear regression analysis was performed on the group (Table 1).

## References

[B1] Akram H. Miller S. Lagrata S. Hyam J. Jahanshahi M. Hariz M.. (2016). Ventral tegmental area deep brain stimulation for refractory chronic cluster headache. Neurology 86, 1676–1682. 10.1212/WNL.0000000000002632 27029635 PMC4854586

[B2] Anderson R. J. Frye M. A. Abulseoud O. A. Lee K. H. McGillivray J. A. Berk M.. (2012). Deep brain stimulation for treatment-resistant depression: efficacy, safety and mechanisms of action. Neurosci. Biobehav. Rev. 36, 1920–1933. 10.1016/j.neubiorev.2012.06.001 22721950

[B3] Berger B. Verney C. Alvarez C. Vigny A. Helle K. B. (1985). New dopaminergic terminal fields in the motor, visual (area 18b) and retrosplenial cortex in the young and adult rat. Immunocytochemical and catecholamine histochemical analyses. Neuroscience 15, 983–998. 10.1016/0306-4522(85)90248-9 2864660

[B4] Bewernick B. H. Hurlemann R. Matusch A. Kayser S. Grubert C. Hadrysiewicz B.. (2010). Nucleus accumbens deep brain stimulation decreases ratings of depression and anxiety in treatment-resistant depression. Biol. Psychiatry 67, 110–116. 10.1016/j.biopsych.2009.09.013 19914605

[B5] Bewernick B. H. Kayser S. Sturm V. Schlaepfer T. E. (2012). Long-term effects of nucleus accumbens deep brain stimulation in treatment-resistant depression: evidence for sustained efficacy. Neuropsychopharmacology 37, 1975–1985. 10.1038/npp.2012.44 22473055 PMC3398749

[B6] Björklund A. Dunnett S. B. (2007). Dopamine neuron systems in the brain: an update. Trends Neurosci. 30, 194–202. 10.1016/j.tins.2007.03.006 17408759

[B7] Bregman T. Reznikov R. Diwan M. Raymond R. Butson C. R. Nobrega J. N. (2015). Antidepressant-like effects of medial forebrain bundle deep brain stimulation in rats are not associated with accumbens dopamine release. Brain Stimul. 8, 708–713. 10.1016/j.brs.2015.02.007 25835354

[B8] Brody A. L. Saxena S. Stoessel P. Gillies L. A. Fairbanks L. A. Alborzian S.. (2001). Regional brain metabolic changes in patients with major depression treated with either paroxetine or interpersonal therapy: preliminary findings. Arch. Gen. Psychiatry 58, 631–640. 10.1001/archpsyc.58.7.631 11448368

[B9] Cleary D. R. Ozpinar A. Raslan A. M. Ko A. L. (2015). Deep brain stimulation for psychiatric disorders: where we are now. Neurosurg. Focus 38, E2. 10.3171/2015.3.FOCUS1546 26030702

[B10] D'Ardenne K. McClure S. M. Nystrom L. E. Cohen J. D. (2008). BOLD responses reflecting dopaminergic signals in the human ventral tegmental area. Science 319, 1264–1267. 10.1126/science.1150605 18309087

[B11] Dichter G. S. Damiano C. A. Allen J. A. (2012). Reward circuitry dysfunction in psychiatric and neurodevelopmental disorders and genetic syndromes: animal models and clinical findings. J. Neurodev. Disord. 4:19. 10.1186/1866-1955-4-19 22958744 PMC3464940

[B12] Dougherty D. D. Rezai A. R. Carpenter L. L. Howland R. H. Bhati M. T. O'Reardon J. P.. (2015). A randomized sham-controlled trial of deep brain stimulation of the ventral capsule/ventral striatum for chronic treatment-resistant depression. Biol. Psychiatry 78, 240–248. 10.1016/j.biopsych.2014.11.023 25726497

[B13] Drevets W. C. Price J. L. Furey M. L. (2008). Brain structural and functional abnormalities in mood disorders: implications for neurocircuitry models of depression. Brain Struct. Funct. 213, 93–118. 10.1007/s00429-008-0189-x 18704495 PMC2522333

[B14] Drevets W. C. Raichle M. E. (1992). Neuroanatomical circuits in depression: implications for treatment mechanisms. Psychopharmacol. Bull. 28, 261–274. 1480730

[B15] Fallon J. H. Moore R. Y. (1978). Catecholamine innervation of the basal forebrain. IV. Topography of the dopamine projection to the basal forebrain and neostriatum. J. Comp. Neurol. 180, 545–580. 10.1002/cne.901800310 659674

[B16] Félix B. Léger M. E. Albe-Fessard D. Marcilloux J. C. Rampin O. Laplace J. P. (1999). Stereotaxic atlas of the pig brain. Brain Res. Bull. 49, 1–137. 10.1016/S0361-9230(99)00012-X 10466025

[B17] Friedman A. Frankel M. Flaumenhaft Y. Merenlender A. Pinhasov A. Feder Y.. (2009). Programmed acute electrical stimulation of ventral tegmental area alleviates depressive-like behavior. Neuropsychopharmacology 34, 1057–1066. 10.1038/npp.2008.177 18843267

[B18] Furlanetti L. L. Coenen V. A. Döbrössy M. D. (2016). Ventral tegmental area dopaminergic lesion-induced depressive phenotype in the rat is reversed by deep brain stimulation of the medial forebrain bundle. Behav. Brain Res. 299, 132–140. 10.1016/j.bbr.2015.11.036 26657994

[B19] Gazit T. Friedman A. Lax E. Samuel M. Zahut R. Katz M.. (2015). Programmed deep brain stimulation synchronizes VTA gamma band field potential and alleviates depressive-like behavior in rats. Neuropharmacology 91, 135–141. 10.1016/j.neuropharm.2014.12.003 25497452

[B20] Gibson W. S. Jo H. J. Testini P. Cho S. Felmlee J. P. Welker K. M.. (2016a). Functional correlates of the therapeutic and adverse effects evoked by thalamic stimulation for essential tremor. Brain 139, 2198–2210. 10.1093/brain/aww145 27329768 PMC4958905

[B21] Gibson W. S. Ross E. K. Han S. R. Van Gompel J. J. Min H. K. Lee K. H. (2016b). Anterior thalamic deep brain stimulation: functional activation patterns in a large animal model. Brain Stimul. 9, 770–773. 10.1016/j.brs.2016.04.012 27160467 PMC5007150

[B22] Goldapple K. Segal Z. Garson C. Lau M. Bieling P. Kennedy S.. (2004). Modulation of cortical-limbic pathways in major depression: treatment-specific effects of cognitive behavior therapy. Arch. Gen. Psychiatry 61, 34–41. 10.1001/archpsyc.61.1.34 14706942

[B23] Hadley J. A. Nenert R. Kraguljac N. V. Bolding M. S. White D. M. Skidmore F. M.. (2014). Ventral tegmental area/midbrain functional connectivity and response to antipsychotic medication in schizophrenia. Neuropsychopharmacology 39, 1020–1030. 10.1038/npp.2013.305 24165885 PMC3924537

[B24] Hasler G. Fromm S. Carlson P. J. Luckenbaugh D. A. Waldeck T. Geraci M.. (2008). Neural response to catecholamine depletion in unmedicated subjects with major depressive disorder in remission and healthy subjects. Arch. Gen. Psychiatry 65, 521–531. 10.1001/archpsyc.65.5.521 18458204 PMC2676777

[B25] Helbing C. Brocka M. Scherf T. Lippert M. T. Angenstein F. (2016). The role of the mesolimbic dopamine system in the formation of blood-oxygen-level dependent responses in the medial prefrontal/anterior cingulate cortex during high-frequency stimulation of the rat perforant pathway. J. Cereb. Blood Flow Metab. 36, 2177–2193. 10.1177/0271678X15615535 26661229 PMC5363663

[B26] Hernandez G. Hamdani S. Rajabi H. Conover K. Stewart J. Arvanitogiannis A.. (2006). Prolonged rewarding stimulation of the rat medial forebrain bundle: neurochemical and behavioral consequences. Behav. Neurosci. 120, 888–904. 10.1037/0735-7044.120.4.888 16893295

[B27] Joel D. Weiner I. (1997). The connections of the primate subthalamic nucleus: indirect pathways and the open-interconnected scheme of basal ganglia-thalamocortical circuitry. Brain Res. Brain Res. Rev. 23, 62–78. 10.1016/S0165-0173(96)00018-5 9063587

[B28] Kennedy S. H. Evans K. R. Krüger S. Mayberg H. S. Meyer J. H. McCann S.. (2001). Changes in regional brain glucose metabolism measured with positron emission tomography after paroxetine treatment of major depression. Am. J. Psychiatry 158, 899–905. 10.1176/appi.ajp.158.6.899 11384897

[B29] Kim J. P. Min H. K. Knight E. J. Duffy P. S. Abulseoud O. A. Marsh M. P.. (2013). Centromedian-parafascicular deep brain stimulation induces differential functional inhibition of the motor, associative, and limbic circuits in large animals. Biol. Psychiatry 74, 917–926. 10.1016/j.biopsych.2013.06.024 23993641 PMC3910443

[B30] Knight E. J. Min H. K. Hwang S. C. Marsh M. P. Paek S. Kim I.. (2013). Nucleus accumbens deep brain stimulation results in insula and prefrontal activation: a large animal FMRI study. PLoS ONE 8:e56640. 10.1371/journal.pone.0056640 23441210 PMC3575484

[B31] Knight E. J. Testini P. Min H. K. Gibson W. S. Gorny K. R. Favazza C. P.. (2015). Motor and nonmotor circuitry activation induced by subthalamic nucleus deep brain stimulation in patients with parkinson disease: intraoperative functional magnetic resonance imaging for deep brain stimulation. Mayo Clin. Proc. 90, 773–785. 10.1016/j.mayocp.2015.03.022 26046412 PMC4469128

[B32] Kunori N. Kajiwara R. Takashima I. (2014). Voltage-sensitive dye imaging of primary motor cortex activity produced by ventral tegmental area stimulation. J. Neurosci. 34, 8894–8903. 10.1523/JNEUROSCI.5286-13.2014 24966388 PMC6608198

[B33] Kwon H. G. Jang S. H. (2014). Differences in neural connectivity between the substantia nigra and ventral tegmental area in the human brain. Front. Hum. Neurosci. 8:41. 10.3389/fnhum.2014.00041 24567711 PMC3915097

[B34] Leech R. Sharp D. J. (2014). The role of the posterior cingulate cortex in cognition and disease. Brain 137, 12–32. 10.1093/brain/awt162 23869106 PMC3891440

[B35] Lozano A. M. Giacobbe P. Hamani C. Rizvi S. J. Kennedy S. H. Kolivakis T. T.. (2012). A multicenter pilot study of subcallosal cingulate area deep brain stimulation for treatment-resistant depression. J. Neurosurg. 116, 315–322. 10.3171/2011.10.JNS102122 22098195

[B36] Lozano A. M. Mayberg H. S. Giacobbe P. Hamani C. Craddock R. C. Kennedy S. H. (2008). Subcallosal cingulate gyrus deep brain stimulation for treatment-resistant depression. Biol. Psychiatry 64, 461–467. 10.1016/j.biopsych.2008.05.034 18639234

[B37] Malone D. A. Jr. Dougherty D. D. Rezai A. R. Carpenter L. L. Friehs G. M. Eskandar E. N.. (2009). Deep brain stimulation of the ventral capsule/ventral striatum for treatment-resistant depression. Biol. Psychiatry 65, 267–275. 10.1016/j.biopsych.2008.08.029 18842257 PMC3486635

[B38] Mayberg H. S. (2003). Modulating dysfunctional limbic-cortical circuits in depression: towards development of brain-based algorithms for diagnosis and optimised treatment. Br. Med. Bull. 65, 193–207. 10.1093/bmb/65.1.193 12697626

[B39] Mayberg H. S. Brannan S. K. Tekell J. L. Silva J. A. Mahurin R. K. McGinnis S.. (2000). Regional metabolic effects of fluoxetine in major depression: serial changes and relationship to clinical response. Biol. Psychiatry 48, 830–843. 10.1016/S0006-3223(00)01036-2 11063978

[B40] Mayberg H. S. Lozano A. M. Voon V. McNeely H. E. Seminowicz D. Hamani C.. (2005). Deep brain stimulation for treatment-resistant depression. Neuron 45, 651–660. 10.1016/j.neuron.2005.02.014 15748841

[B41] McIntyre C. C. Hahn P. J. (2010). Network perspectives on the mechanisms of deep brain stimulation. Neurobiol. Dis. 38, 329–337. 10.1016/j.nbd.2009.09.022 19804831 PMC2862840

[B42] Min H. K. Hwang S. C. Marsh M. P. Kim I. Knight E. Striemer B.. (2012). Deep brain stimulation induces BOLD activation in motor and non-motor networks: an fMRI comparison study of STN and EN/GPi DBS in large animals. Neuroimage 63, 1408–1420. 10.1016/j.neuroimage.2012.08.006 22967832 PMC3487590

[B43] Min H. K. Ross E. K. Jo H. J. Cho S. Settell M. L. Jeong J. H.. (2016). Dopamine release in the nonhuman primate caudate and putamen depends upon site of stimulation in the subthalamic nucleus. J. Neurosci. 36, 6022–6029. 10.1523/JNEUROSCI.0403-16.2016 27251623 PMC4887566

[B44] Moro E. Esselink R. J. Xie J. Hommel M. Benabid A. L. Pollak P. (2002). The impact on Parkinson's disease of electrical parameter settings in STN stimulation. Neurology 59, 706–713. 10.1212/WNL.59.5.706 12221161

[B45] Nakahara D. Fuchikami K. Ozaki N. Iwasaki T. Nagatsu T. (1992). Differential effect of self-stimulation on dopamine release and metabolism in the rat medial frontal cortex, nucleus accumbens and striatum studied by *in vivo* microdialysis. Brain Res. 574, 164–170. 10.1016/0006-8993(92)90813-O 1638391

[B46] Nakahara D. Nakamura M. Furukawa H. Furuno N. (2000). Intracranial self-stimulation increases differentially *in vivo* hydroxylation of tyrosine but similarly *in vivo* hydroxylation of tryptophan in rat medial prefrontal cortex, nucleus accumbens and striatum. Brain Res. 864, 124–129. 10.1016/S0006-8993(00)02166-1 10793194

[B47] Neumeister A. Nugent A. C. Waldeck T. Geraci M. Schwarz M. Bonne O.. (2004). Neural and behavioral responses to tryptophan depletion in unmedicated patients with remitted major depressive disorder and controls. Arch. Gen. Psychiatry 61, 765–773. 10.1001/archpsyc.61.8.765 15289275

[B48] Oades R. D. Halliday G. M. (1987). Ventral tegmental (A10) system: neurobiology. 1. Anatomy and connectivity. Brain Res. 434, 117–165. 10.1016/0165-0173(87)90011-7 3107759

[B49] Paek S. B. Min H. K. Kim I. Knight E. J. Baek J. J. Bieber A. J.. (2015). Frequency-dependent functional neuromodulatory effects on the motor network by ventral lateral thalamic deep brain stimulation in swine. Neuroimage 105, 181–188. 10.1016/j.neuroimage.2014.09.064 25451479 PMC4316813

[B50] Parent A. Hazrati L. N. (1995). Functional anatomy of the basal ganglia. II. The place of subthalamic nucleus and external pallidum in basal ganglia circuitry. Brain Res. Brain Res. Rev. 20, 128–154. 10.1016/0165-0173(94)00008-D 7711765

[B51] Pioli E. Y. Meissner W. Sohr R. Gross C. E. Bezard E. Bioulac B. H. (2008). Differential behavioral effects of partial bilateral lesions of ventral tegmental area or substantia nigra pars compacta in rats. Neuroscience 153, 1213–1224. 10.1016/j.neuroscience.2008.01.084 18455318

[B52] Pochon J. B. Levy R. Fossati P. Lehericy S. Poline J. B. Pillon B.. (2002). The neural system that bridges reward and cognition in humans: an fMRI study. Proc. Natl. Acad. Sci. U.S.A. 99, 5669–5674. 10.1073/pnas.082111099 11960021 PMC122829

[B53] Ross E. K. Kim J. P. Settell M. L. Han S. R. Blaha C. D. Min H. K.. (2016). Fornix deep brain stimulation circuit effect is dependent on major excitatory transmission via the nucleus accumbens. Neuroimage 128, 138–148. 10.1016/j.neuroimage.2015.12.056 26780572 PMC4764383

[B54] Russo S. J. Nestler E. J. (2013). The brain reward circuitry in mood disorders. Nat. Rev. Neurosci. 14, 609–625. 10.1038/nrn3381 23942470 PMC3867253

[B55] Saikali S. Meurice P. Sauleau P. Eliat P. A. Bellaud P. Randuineau G.. (2010). A three-dimensional digital segmented and deformable brain atlas of the domestic pig. J. Neurosci. Methods 192, 102–109. 10.1016/j.jneumeth.2010.07.041 20692291

[B56] Schlaepfer T. E. Bewernick B. H. Kayser S. Mädler B. Coenen V. A. (2013). Rapid effects of deep brain stimulation for treatment-resistant major depression. Biol. Psychiatry 73, 1204–1212. 10.1016/j.biopsych.2013.01.034 23562618

[B57] Singh M. K. Gotlib I. H. (2014). The neuroscience of depression: implications for assessment and intervention. Behav. Res. Ther. 62, 60–73. 10.1016/j.brat.2014.08.008 25239242 PMC4253641

