# Supplementary figures and images for: Functional Circuitry Effect of Ventral Tegmental Area Deep Brain Stimulation: Imaging and Neurochemical Evidence of Mesocortical and Mesolimbic Pathway Modulation
Source: Front Neurosci. 2017 Mar 3;11:104. doi: 10.3389/fnins.2017.00104 (PMC5334355; doi:10.3389/fnins.2017.00104)

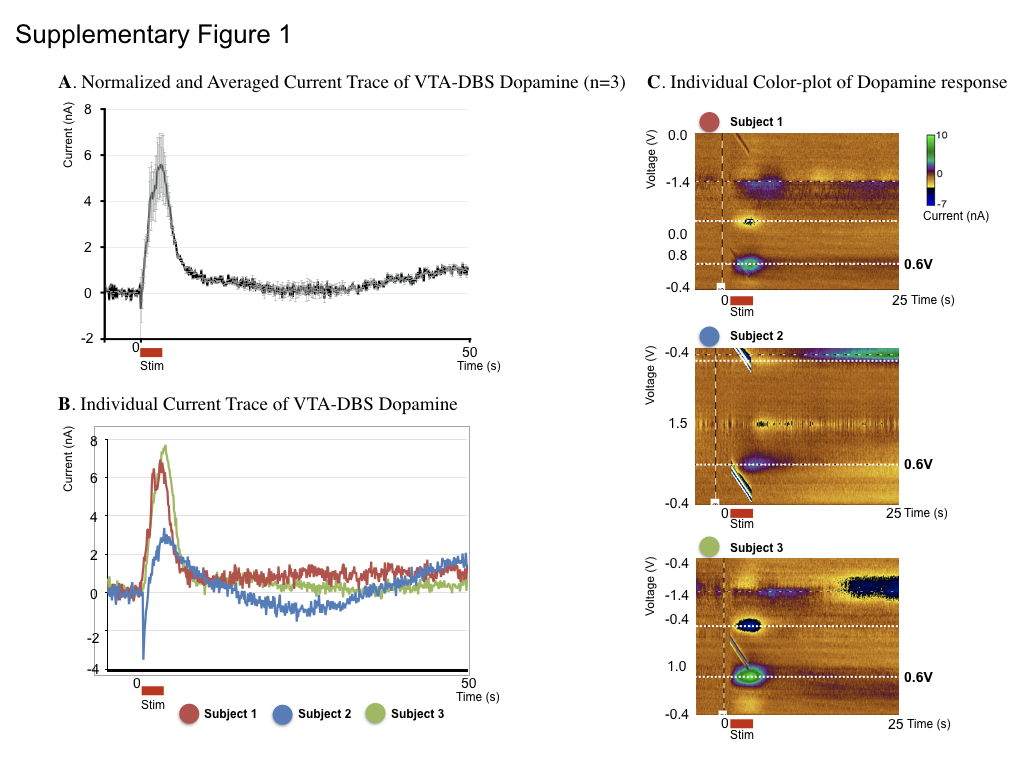

Supplement: Supplementary Figure 1 — Individual dopamine results. [file Image1.TIFF]

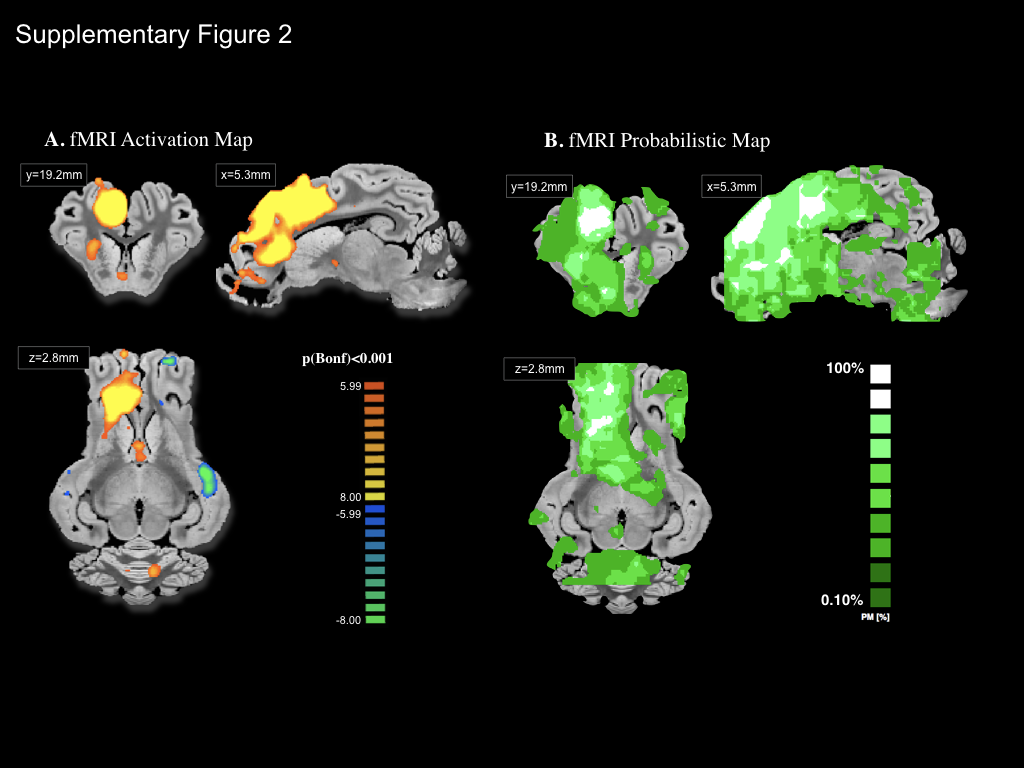

Supplement: Supplementary Figure 2 — To check individual variability, probabilistic map included made with individual FDR < 0.05 activation maps. [file Image2.tiff]
