# Supplementary material for: Functional Circuitry Effect of Ventral Tegmental Area Deep Brain Stimulation: Imaging and Neurochemical Evidence of Mesocortical and Mesolimbic Pathway Modulation
Source: Front Neurosci. 2017 Mar 3;11:104. doi: 10.3389/fnins.2017.00104 (PMC5334355; doi:10.3389/fnins.2017.00104)
Supplement: Supplementary Table 1 — Summary of coefficients for individual linear regression analysis. The table shows the regression coefficients of the voltage changes (1, 2, and 3 V) on the percent change of BOLD peaks by region-of-interests (ROIs) for each of the four subjects. Three parameters (slope, intercept, and coefficient determination—R2) were estimated separately for each subject. The R-square value of the individual indicates the proportion of the within-subject variance in BOLD % change, predicted from the voltage changes. R2 demonstrates the tendency of inter-subject variability in voltage-dependent effects. Linear regression analysis was performed on the group (Table 1). [file Table1.pdf]

Supplementary Table 1

	Sub. 1			Sub. 2			Sub. 3			Sub. 4		
**Regions**	**Slope**	**Intercept**	**R^2**	**Slope**	**Intercept**	**R^2**	**Slope**	**Intercept**	**R^2**	**Slope**	**Intercept**	**R^2**
**APFC**	0.37	-0.46	0.97	1.14	-1.00	0.86	-0.06	0.09	0.81	0.39	0.75	0.72
**CD**	0.23	0.99	0.25	0.62	0.51	1.00	0.56	-0.20	0.37	0.39	1.91	0.89
**DLPFC**	0.66	-0.39	0.36	0.68	-0.72	0.31	0.66	-0.50	0.75	0.47	1.87	0.95
**NAc**	0.61	0.06	0.83	0.91	-0.26	0.87	0.94	-0.05	0.45	0.08	2.23	0.23
**PIMC**	0.83	-0.54	0.81	0.91	-0.65	0.78	0.89	-1.47	0.92	0.21	1.92	0.48
**PEMC**	0.53	-0.53	1.00	1.00	-0.87	0.60	0.80	-0.52	0.78	-0.03	1.93	0.05
**PSSC**	0.34	-0.33	1.00	1.00	0.51	0.84	0.92	-0.07	0.00	0.39	0.37	0.69
**PT**	0.09	0.96	0.89	0.94	-0.19	0.96	0.98	-0.01	0.60	-0.20	1.43	0.99
